# *N*-[2-(4-Bromo­phen­yl)-5-methyl-4-oxo-1,3-thia­zolidin-3-yl]pyridine-3-carboxamide

**DOI:** 10.1107/S1600536811009603

**Published:** 2011-03-19

**Authors:** Mehmet Akkurt, Ísmail Çelik, Hale Demir, Sumru Özkırımlı, Orhan Büyükgüngör

**Affiliations:** a Department of Physics, Faculty of Sciences, Erciyes University, 38039 Kayseri, Turkey; b Department of Physics, Faculty of Arts and Sciences, Cumhuriyet University, 58140 Sivas, Turkey; c Department of Pharmaceutical Chemistry, Faculty of Pharmacy, Istanbul University, 34116 Beyazit, Istanbul, Turkey; d Department of Physics, Faculty of Arts and Sciences, Ondokuz Mayıs University, 55139 Samsun, Turkey

## Abstract

In the title compound, C_16_H_14_BrN_3_O_2_S, the atoms of the 1,3-thia­zolidine group, except for the N and the C atoms attached to the bromo­benzene ring, are disordered over two sets of sites with occupancies of 0.605 (13) and 0.395 (13). The benzene and pyridine rings make a dihedral angle of 86.2 (2)°. In the crystal, mol­ecules are linked by inter­molecular N—H⋯N and C—H⋯O hydrogen bonds, forming a three-dimensional network. Furthermore, there is a π–π stacking inter­action [centroid–centroid distance = 3.758 (2) Å] between the pyridine and benzene rings.

## Related literature

For the diverse pharmacological properties of pyridine-3-carboxamides, see: Abdel-Alim *et al.* (2005 ▶); Girgis *et al.* (2006 ▶); Slominska *et al.* (2008 ▶); Spanka *et al.* (2010 ▶); activities. For the pharmacological properties of 4-thia­zolidinone deriv­atives, see: Vigorita *et al.* (1992 ▶); Barreca *et al.* (2003 ▶); Rao *et al.* (2004 ▶); Jacop & Kutty (2004 ▶); Kalia *et al.* (2007 ▶).
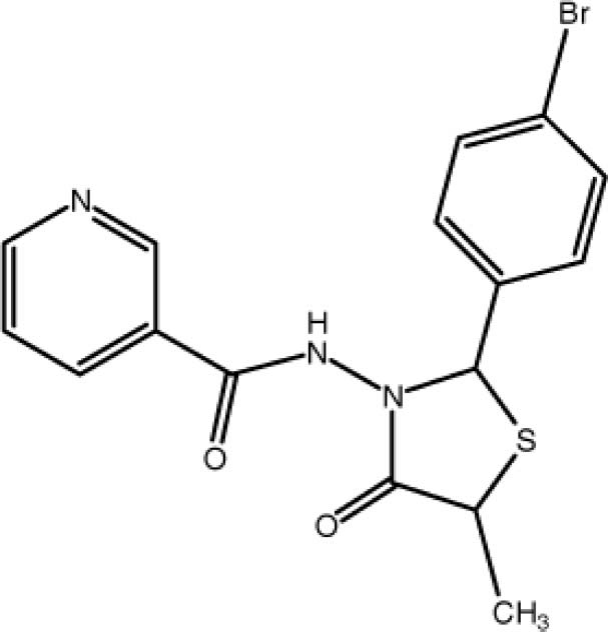


## Experimental

### 

#### Crystal data

C_16_H_14_BrN_3_O_2_S
*M* *_r_* = 392.27
Tetragonal, 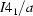

*a* = 24.5799 (8) Å
*c* = 10.9601 (6) Å
*V* = 6621.8 (5) Å^3^
*Z* = 16
Mo *K*α radiation
μ = 2.62 mm^−1^
*T* = 296 K
0.37 × 0.30 × 0.28 mm

#### Data collection

Stoe IPDS 2 diffractometer
Absorption correction: integration (*X-RED32*; Stoe & Cie, 2002 ▶) *T*_min_ = 0.444, *T*_max_ = 0.527
13377 measured reflections
3644 independent reflections
1834 reflections with *I* > 2σ(*I*)
*R*_int_ = 0.055

#### Refinement

*R*[*F* ^2^ > 2σ(*F* ^2^)] = 0.058
*wR*(*F* ^2^) = 0.119
*S* = 1.02
3644 reflections
259 parameters
13 restraints
H atoms treated by a mixture of independent and constrained refinement
Δρ_max_ = 0.39 e Å^−3^
Δρ_min_ = −0.34 e Å^−3^

### 

Data collection: *X-AREA* (Stoe & Cie, 2002 ▶); cell refinement: *X-AREA*; data reduction: *X-RED32* (Stoe & Cie, 2002 ▶); program(s) used to solve structure: *SIR97* (Altomare *et al.*, 1999 ▶); program(s) used to refine structure: *SHELXL97* (Sheldrick, 2008 ▶); molecular graphics: *ORTEP-3* (Farrugia, 1997 ▶); software used to prepare material for publication: *WinGX* (Farrugia, 1999 ▶).

## Supplementary Material

Crystal structure: contains datablocks global, I. DOI: 10.1107/S1600536811009603/om2414sup1.cif

Structure factors: contains datablocks I. DOI: 10.1107/S1600536811009603/om2414Isup2.hkl

Additional supplementary materials:  crystallographic information; 3D view; checkCIF report

## Figures and Tables

**Table 1:** Hydrogen-bond geometry (Å, °)

*D*—H⋯*A*	*D*—H	H⋯*A*	*D*⋯*A*	*D*—H⋯*A*
N2—H*N*2⋯N3^i^	0.861 (19)	2.05 (2)	2.899 (5)	167 (4)
C1—H1⋯O2^ii^	0.93	2.47	3.295 (5)	149
C15—H15⋯O1*A*^iii^	0.93	2.38	3.062 (16)	130

Symmetry codes: (i) 
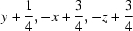
; (ii) 
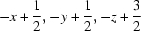
; (iii) 
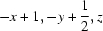
.

**Fig. 1.**
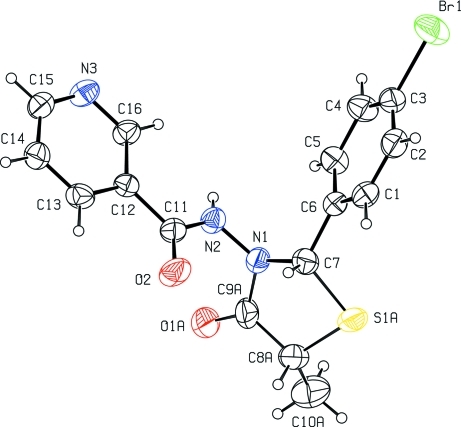
The title molecule with the atom numbering scheme. Displacement ellipsoids for non-H atoms are drawn at the 30% probability level. Only the major component of the disorder is shown.

**Fig. 2.**
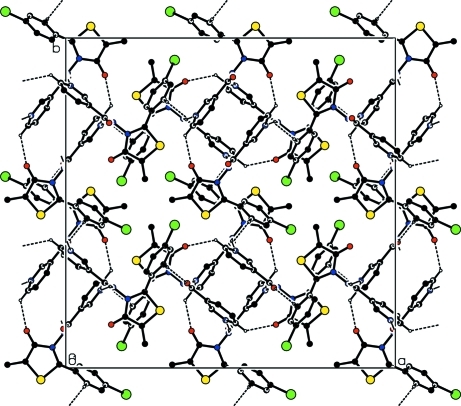
The packing and hydrogen bonding interactions viewed down the c axis. All hydrogen atoms not involved in hydrogen bonding and the minor component of the disorder have been omitted for clarity.

## supplementary crystallographic information

### Comment

Pyridine-3-carboxamides have gained attention because of their diverse pharmacological properties such as anti-inflammatory (Abdel-Alim *et al.*, 2005), anticancer (Girgis *et al.* 2006), cytoprotective (Slominska *et al.*, 2008), and anxiolytic (Spanka *et al.*, 2010) activities. 4-Thiazolidinone derivatives have antineoplastic (Vigorita *et al.*, 1992), HIV-1 RT inhibitory (Barreca *et al.*, 2003; Rao *et al.*, 2004), hypolipidemic (Jacop & Kutty, 2004), and anti-inflammatory (Kalia *et al.*, 2007) activities. In an effort to evaluate bioactive molecules bearing both 4-thiazolidinone and pyridine-3-carboxamide scaffolds together, we synthesized *N*-(thiazolidin-3-yl)pyridine-3-carboxamide derivatives to examine their antiviral and anticancer properties.

In the 1,3-thiazolidine group of the title molecule (Fig. 1), all except the N1 and C7 atoms are disordered. The dihedral angle between the ring planes (S1A/N1/C7/C8A/C9A and S1B/N1/C7/C8B/C9B) formed by the major and minor disorder components is 18.5 (8) °.

The benzene (C1–C6) and pyridine (N3/C12–C16) rings make a dihedral angle of 86.21 (19) ° with each other. The N1—N2—C11—C12, N1—N2—C11—O2, N2—C11—C12—C13 and O2—C11—C12—C13 torsion angles are 180.0 (3), 1.2 (6), 172.2 (3) and -9.0 (6)°, respectively.

The molecular structure has two weak intramolecular C—H···N interactions (Table 1), generating *S*(5) ring motifs, and the crystal structure is stabilized by intermolecular N—H···N and C—H···O hydrogen bonds (Table 1 and Fig. 2). In addition, a π-π stacking interaction is observed [*Cg*3···*Cg*4(1/4 + *y*, 1/4 - *x*, 1/4 + *z*) = 3.758 (2) Å, where *Cg*3 and *Cg*4 are the centroids of the pyridine N3/C12–C16 and benzene C1–C6 rings, respectively].

### Experimental

*N*'-(4-bromobenzylidine)pyridine-3-carbohydrazide (0.01 mol) was reacted with 0.028 mol of 2-mercaptopropanoic acid in anhydrous benzene for 7 h using a Dean-Stark trap. Excess benzene was removed under reduced pressure. The residue was triturated with saturated sodium bicarbonate solution. The separated solid was filtered, washed with water and crystallized from methanol to yield white crystalline *N*-[5-methyl-2-(4-bromophenyl)-4-oxo-1,3-thiazolidin-3-yl]pyridine-3-carboxamide. Yield: 58.92%; m.p.: 466.0–470.0 K. UV (EtOH) λ max: 203.0, 220.6, 262.0 nm. IR (KBr) υ: 1674 (amide C=O), 1727 (thia C=O) cm^-1^; ^1^H-NMR (DMSO-d_6_, 500 MHz): 1.54, 1.55 (3*H*, 2 d, J=7.0 Hz, 6.8 Hz, CH_3_-thia.), 4.12, 4.22 (1*H*, 2q, J=6.8 Hz, 6.8 Hz, H5-thia.), 5.90 (1*H*, s, H2-thia), 7.44–7.46 (2*H*, m, 2-C_6_H_4_-(H2,6)-thia.), 7.49–7.56 (1*H*, m, H5-pyridine), 7.57–7.60 (2*H*, m, 2-C_6_H_4_-(H3,5)-thia.), 8.05–8.09 (1*H*, m, H4-pyridine), 8.72–8.73 (1*H*, m, H6-pyridine), 8.86 (1*H*, 2 t, J=1 Hz, H2-pyridine), 10.94, 10.95 (1*H*, 2 s, CONH) p.p.m.; ESI– (m/*z*, relative abundance): 392.0 ([M—H+2]^-^, 100), 390.01 ([M—H]^-^, 79.69). Analysis calculated for C_16_H_14_BrN_3_O_2_S: C 48.99, H 3.60, N 10.71%. Found: C 49.03, H 3.54, N 10.58%.

### Refinement

The C-bound H atoms were geometrically placed (C—H = 0.93–0.98 Å) and refined as riding with *U*_iso_(H) = 1.2 or 1.5*U*_eq_(C). The N-bound H atom was located from the Fourier synthesis and restrained to 0.86 (2) Å, and refined with *U*_iso_(H) = 1.2*U*_eq_(N). The 1,3-thiazolidine group, except for the N1 and C7 atoms, is disordered over two sites with site occupancies of 0.605 (13) and 0.395 (13). In the last cycles of the refinement, the following values are used for the distance restraints (DFIX): 1.80 (1) Å for the S—C bond, 1.25 (1) Å for C—O, 1.39 (1) Å for C—N and 1.50 (1) Å for C—C

### Crystal data

**Table:**

C_16_H_14_BrN_3_O_2_S	*D*_x_ = 1.574 Mg m^−^^3^
*M**_r_* = 392.27	Mo *K*α radiation, λ = 0.71073 Å
Tetragonal, *I*4_1_/*a*	Cell parameters from 9828 reflections
Hall symbol: -I 4ad	θ = 1.7–27.6°
*a* = 24.5799 (8) Å	µ = 2.62 mm^−^^1^
*c* = 10.9601 (6) Å	*T* = 296 K
*V* = 6621.8 (5) Å^3^	Block, colourless
*Z* = 16	0.37 × 0.30 × 0.28 mm
*F*(000) = 3168	

### Data collection

**Table:**

Stoe IPDS 2 diffractometer	3644 independent reflections
Radiation source: sealed X-ray tube, 12 x 0.4 mm long-fine focus	1834 reflections with *I* > 2σ(*I*)
plane graphite	*R*_int_ = 0.055
Detector resolution: 6.67 pixels mm^-1^	θ_max_ = 27.1°, θ_min_ = 1.7°
ω scans	*h* = −31→21
Absorption correction: integration (*X-RED32*; Stoe & Cie, 2002)	*k* = −31→31
*T*_min_ = 0.444, *T*_max_ = 0.527	*l* = −14→9
13377 measured reflections	

### Refinement

**Table:**

Refinement on *F*^2^	Primary atom site location: structure-invariant direct methods
Least-squares matrix: full	Secondary atom site location: difference Fourier map
*R*[*F*^2^ > 2σ(*F*^2^)] = 0.058	Hydrogen site location: inferred from neighbouring sites
*wR*(*F*^2^) = 0.119	H atoms treated by a mixture of independent and constrained refinement
*S* = 1.02	*w* = 1/[σ^2^(*F*_o_^2^) + (0.046*P*)^2^] where *P* = (*F*_o_^2^ + 2*F*_c_^2^)/3
3644 reflections	(Δ/σ)_max_ < 0.001
259 parameters	Δρ_max_ = 0.39 e Å^−^^3^
13 restraints	Δρ_min_ = −0.34 e Å^−^^3^

### Special details

**Table:**

Geometry. Bond distances, angles *etc*. have been calculated using the rounded fractional coordinates. All su's are estimated from the variances of the (full) variance-covariance matrix. The cell e.s.d.'s are taken into account in the estimation of distances, angles and torsion angles
Refinement. Refinement on *F*^2^ for ALL reflections except those flagged by the user for potential systematic errors. Weighted *R*-factors *wR* and all goodnesses of fit *S* are based on *F*^2^, conventional *R*-factors *R* are based on *F*, with *F* set to zero for negative *F*^2^. The observed criterion of *F*^2^ > σ(*F*^2^) is used only for calculating -*R*-factor-obs *etc*. and is not relevant to the choice of reflections for refinement. *R*-factors based on *F*^2^ are statistically about twice as large as those based on *F*, and *R*-factors based on ALL data will be even larger.

### Fractional atomic coordinates and isotropic or equivalent isotropic displacement parameters (Å^2^)

**Table:**

	*x*	*y*	*z*	*U*_iso_*/*U*_eq_	Occ. (<1)
Br1	0.16970 (2)	0.06678 (2)	0.30959 (7)	0.1147 (3)	
S1A	0.2142 (3)	0.3258 (3)	0.5735 (10)	0.112 (3)	0.605 (13)
O1A	0.3622 (6)	0.3685 (8)	0.529 (2)	0.106 (7)	0.605 (13)
O2	0.36844 (13)	0.25700 (14)	0.7196 (3)	0.0950 (14)	
N1	0.31204 (11)	0.29058 (12)	0.5238 (3)	0.0623 (13)	
N2	0.35645 (13)	0.25553 (13)	0.5165 (3)	0.0595 (11)	
N3	0.48519 (14)	0.14473 (13)	0.4828 (4)	0.0707 (14)	
C1	0.20330 (14)	0.18614 (15)	0.5608 (4)	0.0640 (14)	
C2	0.18240 (16)	0.14118 (16)	0.5027 (5)	0.0730 (16)	
C3	0.19861 (16)	0.12919 (15)	0.3869 (5)	0.0673 (18)	
C4	0.23459 (18)	0.16225 (18)	0.3269 (4)	0.0763 (17)	
C5	0.25484 (16)	0.20793 (16)	0.3847 (4)	0.0653 (16)	
C6	0.23981 (14)	0.22004 (14)	0.5022 (4)	0.0560 (14)	
C7	0.26058 (14)	0.26964 (15)	0.5686 (4)	0.0703 (16)	
C8A	0.2692 (4)	0.3725 (3)	0.5910 (13)	0.075 (4)	0.605 (13)
C9A	0.3197 (5)	0.3447 (3)	0.548 (2)	0.079 (8)	0.605 (13)
C10A	0.2598 (5)	0.4271 (4)	0.5333 (15)	0.125 (7)	0.605 (13)
C11	0.38243 (15)	0.24133 (15)	0.6199 (5)	0.0603 (14)	
C12	0.42975 (14)	0.20345 (14)	0.6039 (4)	0.0510 (14)	
C13	0.46220 (17)	0.19258 (16)	0.7037 (4)	0.0673 (16)	
C14	0.50564 (16)	0.15811 (16)	0.6913 (5)	0.0713 (18)	
C15	0.51542 (16)	0.13554 (16)	0.5804 (5)	0.0663 (16)	
C16	0.44267 (16)	0.17785 (16)	0.4962 (4)	0.0630 (14)	
O1B	0.3661 (9)	0.3633 (10)	0.482 (3)	0.077 (6)	0.395 (13)
C8B	0.2702 (5)	0.3783 (5)	0.5205 (14)	0.063 (5)	0.395 (13)
S1B	0.2171 (4)	0.3287 (4)	0.5249 (11)	0.078 (2)	0.395 (13)
C10B	0.2737 (7)	0.4152 (8)	0.6288 (16)	0.100 (7)	0.395 (13)
C9B	0.3209 (8)	0.3454 (4)	0.508 (3)	0.059 (7)	0.395 (13)
H5	0.27890	0.23070	0.34360	0.0780*	
H7	0.26680	0.25850	0.65330	0.0840*	
H4	0.24530	0.15400	0.24770	0.0910*	
H10A	0.22620	0.44200	0.56260	0.1870*	0.605 (13)
H10B	0.25810	0.42300	0.44630	0.1870*	0.605 (13)
H8A	0.27360	0.37870	0.67870	0.07 (2)*	0.605 (13)
H13	0.45460	0.20850	0.77870	0.0810*	
H14	0.52800	0.15030	0.75750	0.0850*	
H15	0.54510	0.11230	0.57270	0.0800*	
H16	0.42050	0.18400	0.42880	0.0760*	
H1	0.19280	0.19380	0.64040	0.0770*	
HN2	0.3657 (17)	0.2533 (17)	0.4409 (14)	0.0810*	
H10C	0.28920	0.45110	0.55420	0.1870*	0.605 (13)
H2A	0.15730	0.11910	0.54230	0.0870*	
H8B	0.26570	0.40050	0.44690	0.0750*	0.395 (13)
H10D	0.24060	0.43570	0.63580	0.1500*	0.395 (13)
H10E	0.30370	0.43980	0.61860	0.1500*	0.395 (13)
H10F	0.27900	0.39400	0.70120	0.1500*	0.395 (13)

### Atomic displacement parameters (Å^2^)

**Table:**

	*U*^11^	*U*^22^	*U*^33^	*U*^12^	*U*^13^	*U*^23^
Br1	0.1245 (5)	0.0791 (3)	0.1405 (6)	−0.0194 (3)	−0.0237 (4)	−0.0275 (3)
S1A	0.0607 (18)	0.092 (3)	0.183 (8)	−0.0045 (15)	0.027 (3)	−0.069 (3)
O1A	0.066 (5)	0.084 (5)	0.167 (19)	−0.012 (4)	0.003 (7)	−0.028 (8)
O2	0.094 (2)	0.131 (3)	0.060 (2)	0.0395 (19)	0.0025 (17)	−0.029 (2)
N1	0.0498 (17)	0.0572 (18)	0.080 (3)	0.0016 (14)	0.0006 (16)	−0.0031 (17)
N2	0.0555 (18)	0.071 (2)	0.052 (2)	0.0053 (15)	0.0044 (18)	−0.0008 (19)
N3	0.076 (2)	0.074 (2)	0.062 (3)	0.0162 (18)	0.013 (2)	0.0013 (19)
C1	0.060 (2)	0.070 (2)	0.062 (3)	0.0051 (19)	0.014 (2)	−0.001 (2)
C2	0.061 (2)	0.059 (2)	0.099 (4)	−0.0032 (19)	0.004 (3)	0.009 (2)
C3	0.069 (3)	0.055 (2)	0.078 (4)	0.0041 (19)	−0.010 (2)	−0.005 (2)
C4	0.090 (3)	0.082 (3)	0.057 (3)	−0.004 (2)	0.002 (2)	−0.010 (3)
C5	0.070 (3)	0.068 (2)	0.058 (3)	−0.0105 (19)	0.004 (2)	0.003 (2)
C6	0.051 (2)	0.057 (2)	0.060 (3)	0.0035 (17)	0.003 (2)	−0.005 (2)
C7	0.059 (2)	0.077 (3)	0.075 (3)	−0.0095 (18)	0.009 (2)	−0.016 (2)
C8A	0.083 (6)	0.065 (6)	0.076 (10)	0.000 (4)	0.005 (7)	−0.012 (6)
C9A	0.050 (7)	0.076 (8)	0.11 (2)	−0.008 (5)	−0.009 (6)	−0.003 (5)
C10A	0.139 (9)	0.073 (7)	0.162 (16)	0.020 (6)	−0.004 (9)	−0.001 (8)
C11	0.061 (2)	0.067 (2)	0.053 (3)	0.0035 (18)	0.004 (2)	−0.007 (2)
C12	0.057 (2)	0.053 (2)	0.043 (3)	−0.0029 (16)	0.0041 (19)	−0.0006 (18)
C13	0.082 (3)	0.069 (2)	0.051 (3)	0.004 (2)	−0.006 (2)	−0.007 (2)
C14	0.070 (3)	0.066 (2)	0.078 (4)	0.008 (2)	−0.017 (2)	0.000 (3)
C15	0.061 (2)	0.060 (2)	0.078 (4)	0.0060 (18)	0.007 (2)	0.002 (2)
C16	0.070 (2)	0.070 (2)	0.049 (3)	0.012 (2)	0.003 (2)	−0.002 (2)
O1B	0.073 (8)	0.063 (9)	0.096 (14)	−0.008 (6)	0.006 (7)	0.010 (9)
C8B	0.072 (7)	0.063 (8)	0.053 (10)	0.008 (5)	0.001 (8)	0.012 (8)
S1B	0.049 (3)	0.069 (3)	0.115 (6)	0.0113 (17)	0.007 (3)	−0.030 (3)
C10B	0.110 (11)	0.078 (11)	0.112 (15)	−0.013 (8)	−0.001 (10)	−0.020 (11)
C9B	0.069 (12)	0.057 (10)	0.052 (14)	0.006 (8)	−0.001 (7)	−0.004 (6)

### Geometric parameters (Å, °)

**Table:**

Br1—C3	1.891 (4)	C8B—C9B	1.49 (2)
S1A—C7	1.791 (8)	C8B—C10B	1.50 (2)
S1A—C8A	1.784 (12)	C11—C12	1.500 (5)
S1B—C7	1.865 (11)	C12—C13	1.380 (6)
S1B—C8B	1.787 (16)	C12—C16	1.375 (6)
O1A—C9A	1.22 (2)	C13—C14	1.370 (6)
O1B—C9B	1.23 (3)	C14—C15	1.358 (7)
O2—C11	1.209 (6)	C1—H1	0.9300
N1—C9A	1.370 (9)	C2—H2A	0.9300
N1—N2	1.393 (4)	C4—H4	0.9300
N1—C7	1.451 (5)	C5—H5	0.9300
N1—C9B	1.376 (11)	C7—H7	0.9800
N2—C11	1.347 (6)	C8A—H8A	0.9800
N3—C15	1.322 (6)	C8B—H8B	0.9800
N3—C16	1.333 (5)	C10A—H10B	0.9600
N2—HN2	0.861 (19)	C10A—H10C	0.9600
C1—C6	1.383 (5)	C10A—H10A	0.9600
C1—C2	1.375 (6)	C10B—H10F	0.9600
C2—C3	1.363 (7)	C10B—H10D	0.9600
C3—C4	1.369 (6)	C10B—H10E	0.9600
C4—C5	1.382 (6)	C13—H13	0.9300
C5—C6	1.372 (6)	C14—H14	0.9300
C6—C7	1.509 (5)	C15—H15	0.9300
C8A—C9A	1.493 (16)	C16—H16	0.9300
C8A—C10A	1.502 (14)		
			
Br1···C2^i^	3.679 (4)	C14···C1^vii^	3.483 (6)
Br1···C3^i^	3.669 (4)	C15···O1A^iii^	3.062 (16)
S1B···C15^i^	3.651 (12)	C15···O1B^iii^	3.11 (2)
S1B···C14^i^	3.679 (11)	C15···S1B^vii^	3.651 (12)
S1A···H14^i^	3.1300	C16···O1B^vi^	3.23 (3)
S1B···H15^i^	3.0700	C5···H5^ii^	3.0400
S1B···H14^i^	3.1500	C5···HN2	3.01 (4)
S1B···H4^ii^	3.1600	C8B···H10E^viii^	3.0800
O1A···N2	2.78 (2)	C8B···H4^ii^	3.0700
O1A···C15^iii^	3.062 (16)	C10A···H10C^viii^	3.0100
O1A···C11	3.32 (2)	C11···H7	2.9000
O1B···N2	2.69 (2)	C15···HN2^vi^	3.048 (18)
O1B···C11	3.38 (3)	C16···HN2	2.72 (4)
O1B···C16^iv^	3.23 (3)	C16···HN2^vi^	2.88 (3)
O1B···C15^iii^	3.11 (2)	H1···O2^v^	2.4700
O2···N1	2.685 (5)	H1···H7	2.4200
O2···C7	3.141 (5)	HN2···C16	2.72 (4)
O2···C9A	3.102 (15)	HN2···H16	2.1800
O2···C1^v^	3.295 (5)	HN2···C5	3.01 (4)
O2···C9B	3.39 (2)	HN2···N3^iv^	2.05 (2)
O1A···H15^iii^	2.3800	HN2···C15^iv^	3.048 (18)
O1A···H10C	2.7200	HN2···O1B	2.74 (5)
O1B···H10E	2.8500	HN2···C16^iv^	2.88 (3)
O1B···H15^iii^	2.4700	HN2···H5	2.4500
O1B···HN2	2.74 (5)	H4···S1B^ii^	3.1600
O1B···H16^iv^	2.5700	H4···H8B^ii^	2.5300
O2···H1^v^	2.4700	H4···C8B^ii^	3.0700
O2···H7	2.6000	H5···C5^ii^	3.0400
O2···H13	2.5200	H5···HN2	2.4500
N1···O2	2.685 (5)	H5···N1	2.5900
N2···N3^iv^	2.899 (5)	H5···N2	2.7600
N2···O1A	2.78 (2)	H7···C11	2.9000
N2···C5	3.113 (5)	H7···H1	2.4200
N2···O1B	2.69 (2)	H7···O2	2.6000
N3···N2^vi^	2.899 (5)	H7···H7^v^	2.3100
N1···H5	2.5900	H8B···H10E^viii^	2.3400
N2···H16	2.5500	H8B···H4^ii^	2.5300
N2···H5	2.7600	H10B···H10C^viii^	2.2900
N3···HN2^vi^	2.05 (2)	H10C···O1A	2.7200
N3···H16^vi^	2.7400	H10C···H10B^ix^	2.2900
C1···C14^i^	3.483 (6)	H10C···C10A^ix^	3.0100
C1···O2^v^	3.295 (5)	H10E···C8B^ix^	3.0800
C2···C13^i^	3.574 (6)	H10E···O1B	2.8500
C2···Br1^vii^	3.679 (4)	H10E···H8B^ix^	2.3400
C3···Br1^vii^	3.669 (4)	H13···O2	2.5200
C5···N2	3.113 (5)	H14···S1A^vii^	3.1300
C7···O2	3.141 (5)	H14···S1B^vii^	3.1500
C9A···O2	3.102 (15)	H15···O1B^iii^	2.4700
C9B···O2	3.39 (2)	H15···S1B^vii^	3.0700
C11···O1A	3.32 (2)	H15···O1A^iii^	2.3800
C11···O1B	3.38 (3)	H16···N2	2.5500
C13···C13^iii^	3.380 (6)	H16···N3^iv^	2.7400
C13···C2^vii^	3.574 (6)	H16···O1B^vi^	2.5700
C14···S1B^vii^	3.679 (11)	H16···HN2	2.1800
			
C7—S1A—C8A	91.0 (4)	C11—C12—C13	118.4 (4)
C7—S1B—C8B	96.9 (6)	C12—C13—C14	119.5 (4)
N2—N1—C9A	120.3 (6)	C13—C14—C15	118.7 (4)
N2—N1—C9B	118.3 (9)	N3—C15—C14	123.7 (4)
C7—N1—C9A	113.5 (7)	N3—C16—C12	123.7 (4)
C7—N1—C9B	121.9 (9)	C2—C1—H1	120.00
N2—N1—C7	118.9 (3)	C6—C1—H1	120.00
N1—N2—C11	118.9 (3)	C1—C2—H2A	120.00
C15—N3—C16	117.1 (4)	C3—C2—H2A	120.00
C11—N2—HN2	132 (3)	C3—C4—H4	120.00
N1—N2—HN2	108 (3)	C5—C4—H4	120.00
C2—C1—C6	120.8 (4)	C4—C5—H5	120.00
C1—C2—C3	119.7 (4)	C6—C5—H5	120.00
Br1—C3—C4	120.6 (4)	S1A—C7—H7	107.00
Br1—C3—C2	118.9 (3)	N1—C7—H7	106.00
C2—C3—C4	120.5 (4)	C6—C7—H7	106.00
C3—C4—C5	119.7 (4)	S1B—C7—H7	123.00
C4—C5—C6	120.6 (4)	S1A—C8A—H8A	107.00
C1—C6—C7	118.9 (4)	C9A—C8A—H8A	107.00
C1—C6—C5	118.7 (4)	C10A—C8A—H8A	107.00
C5—C6—C7	122.5 (3)	C9B—C8B—H8B	109.00
N1—C7—C6	114.7 (3)	C10B—C8B—H8B	109.00
S1A—C7—C6	115.0 (4)	S1B—C8B—H8B	109.00
S1B—C7—N1	97.9 (4)	C8A—C10A—H10C	109.00
S1B—C7—C6	108.1 (4)	H10A—C10A—H10B	110.00
S1A—C7—N1	107.0 (4)	H10B—C10A—H10C	110.00
S1A—C8A—C9A	107.6 (7)	C8A—C10A—H10B	109.00
C9A—C8A—C10A	113.8 (11)	H10A—C10A—H10C	109.00
S1A—C8A—C10A	114.4 (9)	C8A—C10A—H10A	109.00
S1B—C8B—C10B	115.7 (11)	H10D—C10B—H10F	110.00
C9B—C8B—C10B	110.7 (15)	C8B—C10B—H10D	109.00
S1B—C8B—C9B	104.1 (9)	C8B—C10B—H10E	109.00
N1—C9A—C8A	113.0 (8)	C8B—C10B—H10F	110.00
O1A—C9A—C8A	123.3 (11)	H10D—C10B—H10E	109.00
O1A—C9A—N1	123.6 (14)	H10E—C10B—H10F	110.00
N1—C9B—C8B	112.8 (14)	C14—C13—H13	120.00
O1B—C9B—C8B	125.6 (15)	C12—C13—H13	120.00
O1B—C9B—N1	121.5 (17)	C15—C14—H14	121.00
O2—C11—N2	122.9 (4)	C13—C14—H14	121.00
O2—C11—C12	121.6 (4)	N3—C15—H15	118.00
N2—C11—C12	115.5 (4)	C14—C15—H15	118.00
C13—C12—C16	117.3 (3)	C12—C16—H16	118.00
C11—C12—C16	124.3 (4)	N3—C16—H16	118.00
			
C8A—S1A—C7—N1	−26.3 (7)	C2—C3—C4—C5	0.4 (6)
C8A—S1A—C7—C6	−154.9 (6)	Br1—C3—C4—C5	179.4 (3)
C7—S1A—C8A—C9A	19.9 (12)	C3—C4—C5—C6	0.9 (6)
C7—S1A—C8A—C10A	147.4 (10)	C4—C5—C6—C7	−179.6 (4)
C7—N1—N2—C11	−75.1 (4)	C4—C5—C6—C1	−1.1 (6)
C9A—N1—N2—C11	73.1 (11)	C5—C6—C7—N1	−24.1 (5)
C9A—N1—C7—S1A	26.8 (10)	C5—C6—C7—S1A	100.6 (5)
N2—N1—C7—C6	−54.3 (5)	C1—C6—C7—N1	157.4 (3)
C9A—N1—C7—C6	155.5 (9)	C1—C6—C7—S1A	−77.9 (5)
C7—N1—C9A—C8A	−12.1 (17)	C10A—C8A—C9A—N1	−136.2 (13)
N2—N1—C9A—O1A	23 (3)	S1A—C8A—C9A—N1	−8.3 (18)
C7—N1—C9A—O1A	172.4 (18)	C10A—C8A—C9A—O1A	39 (3)
N2—N1—C9A—C8A	−161.9 (10)	S1A—C8A—C9A—O1A	167 (2)
N2—N1—C7—S1A	177.0 (4)	N2—C11—C12—C13	172.2 (3)
N1—N2—C11—O2	1.2 (6)	O2—C11—C12—C13	−9.0 (6)
N1—N2—C11—C12	180.0 (3)	O2—C11—C12—C16	169.8 (4)
C16—N3—C15—C14	−0.5 (6)	N2—C11—C12—C16	−9.0 (5)
C15—N3—C16—C12	1.6 (6)	C16—C12—C13—C14	1.0 (6)
C2—C1—C6—C7	178.5 (3)	C11—C12—C16—N3	179.2 (4)
C2—C1—C6—C5	0.0 (6)	C11—C12—C13—C14	180.0 (4)
C6—C1—C2—C3	1.3 (6)	C13—C12—C16—N3	−1.9 (6)
C1—C2—C3—C4	−1.5 (6)	C12—C13—C14—C15	−0.1 (6)
C1—C2—C3—Br1	179.5 (3)	C13—C14—C15—N3	−0.2 (6)

Symmetry codes: (i) −*y*+1/4, *x*−1/4, *z*−1/4; (ii) −*x*+1/2, −*y*+1/2, −*z*+1/2; (iii) −*x*+1, −*y*+1/2, *z*; (iv) *y*+1/4, −*x*+3/4, −*z*+3/4; (v) −*x*+1/2, −*y*+1/2, −*z*+3/2; (vi) −*y*+3/4, *x*−1/4, −*z*+3/4; (vii) *y*+1/4, −*x*+1/4, *z*+1/4; (viii) *y*−1/4, −*x*+3/4, *z*−1/4; (ix) −*y*+3/4, *x*+1/4, *z*+1/4.

### Hydrogen-bond geometry (Å, °)

**Table:**

*D*—H···*A*	*D*—H	H···*A*	*D*···*A*	*D*—H···*A*
N2—HN2···N3^iv^	0.861 (19)	2.05 (2)	2.899 (5)	167 (4)
C1—H1···O2^v^	0.93	2.47	3.295 (5)	149
C5—H5···N1	0.93	2.59	2.903 (5)	100
C15—H15···O1A^iii^	0.93	2.38	3.062 (16)	130
C16—H16···N2	0.93	2.55	2.861 (5)	100

Symmetry codes: (iv) *y*+1/4, −*x*+3/4, −*z*+3/4; (v) −*x*+1/2, −*y*+1/2, −*z*+3/2; (iii) −*x*+1, −*y*+1/2, *z*.

## References

[bb1] Abdel-Alim, A. M., El-Shorbagi, A. A., Abdel-Mothy, S. G. & Abdel-Allah, H. H. M. (2005). *Arch. Pharm. Res.* **28**, 637–647. 10.1007/BF02969351 16042070

[bb2] Altomare, A., Burla, M. C., Camalli, M., Cascarano, G. L., Giacovazzo, C., Guagliardi, A., Moliterni, A. G. G., Polidori, G. & Spagna, R. (1999). *J. Appl. Cryst.* **32**, 115–119.

[bb3] Barreca, M. L., Chimirri, A., De Clercq, E., De Luca, L., Monforte, A. M., Monforte, P., Rao, A. & Zappala, M. (2003). *Farmaco*, **58**, 259–263. 10.1016/S0014-827X(03)00024-7 12620421

[bb4] Farrugia, L. J. (1997). *J. Appl. Cryst.* **30**, 565.

[bb5] Farrugia, L. J. (1999). *J. Appl. Cryst.* **32**, 837–838.

[bb6] Girgis, A. S., Hosni, H. M. & Barsoum, F. F. (2006). *Bioorg. Med. Chem.* **14**, 4466–4476. 10.1016/j.bmc.2006.02.031 16524735

[bb7] Jacop, J. & Kutty, G. N. (2004). *Indian Drugs*, **41**, 76–79.

[bb8] Kalia, R., Rao, C. M. & Kutty, N. G. (2007). *Arzneim. Forsch. (Drug Res.)*, **57**, 616–622. 10.1055/s-0031-1296657 17966761

[bb9] Rao, A., Balzarini, J., Carbone, A., Chimirri, A., De Clercq, E., Monforte, A. M., Monforte, P., Pannecouque, C. & Zappala, M. (2004). *Antiviral Res.* **63**, 79–84. 10.1016/j.antiviral.2004.03.004 15302136

[bb10] Sheldrick, G. M. (2008). *Acta Cryst.* A**64**, 112–122. 10.1107/S0108767307043930 18156677

[bb11] Slominska, E. M., Yuen, A., Osman, L., Gebicki, J., Yacoub, M. H. & Smolenski, R. T. (2008). *Nucleoside Nucleotides Nucleic Acids*, **27**, 863-866. 10.1080/15257770802146528 18600553

[bb12] Spanka, C., Glatthar, R., Desrayaud, S., Fendt, M., Orain, D., Troxler, T. & Vranesic, I. (2010). *Bioorg. Med. Chem. Lett.* **20**, 184–188. 10.1016/j.bmcl.2009.11.001 19931453

[bb13] Stoe & Cie (2002). *X-AREA* and *X-RED32* Stoe & Cie, Darmstadt, Germany.

[bb14] Vigorita, M. G., Basile, M., Zappala, C., Gabbrielli, G. & Pizzimenti, F. (1992). *Farmaco*, **47**, 893–906. 1388607

